# Nutritional Preconditioning in Cancer Treatment in Relation to DNA Damage and Aging

**DOI:** 10.1146/annurev-cancerbio-060820-090737

**Published:** 2020-12-02

**Authors:** Winnie M.C. van den Boogaard, Marry M. van den Heuvel-Eibrink, Jan H.J. Hoeijmakers, Wilbert P. Vermeij

**Affiliations:** 1 Genome Instability and Nutrition Research Group, Princess Máxima Center for Pediatric Oncology, 3584 CS Utrecht, The Netherlands;; 2 Oncode Institute, 3521 AL Utrecht, The Netherlands; 3 Pediatric Oncology Translational Research Group, Princess Máxima Center for Pediatric Oncology, 3584 CS Utrecht, The Netherlands; 4 Department of Molecular Genetics, Erasmus University Medical Center, 3015 GD Rotterdam, The Netherlands;; 5 CECAD Forschungszentrum, University of Cologne, 50931 Cologne, Germany

**Keywords:** DNA damage repair, chemotherapy, cancer, aging, dietary restriction, fasting

## Abstract

Dietary restriction (DR) is the most successful nutritional intervention for extending lifespan and preserving health in numerous species. Reducing food intake triggers a protective response that shifts energy resources from growth to maintenance and resilience mechanisms. This so-called survival response has been shown to particularly increase life- and health span and decrease DNA damage in DNA repair–deficient mice exhibiting accelerated aging. Accumulation of DNA damage is the main cause of aging, but also of cancer. Moreover, radiotherapies and most chemotherapies are based on damaging DNA, consistent with their ability to induce toxicity and accelerate aging. Since fasting and DR decrease DNA damage and its effects, nutritional preconditioning holds promise for improving (cancer) therapy and preventing short- and long-term side effects of anticancer treatments. This review provides an overview of the link between aging and cancer, highlights important preclinical studies applying such nutritional preconditioning, and summarizes the first clinical trials implementing nutritional preconditioning in cancer treatment.

## INTRODUCTION

**Figure 1:**
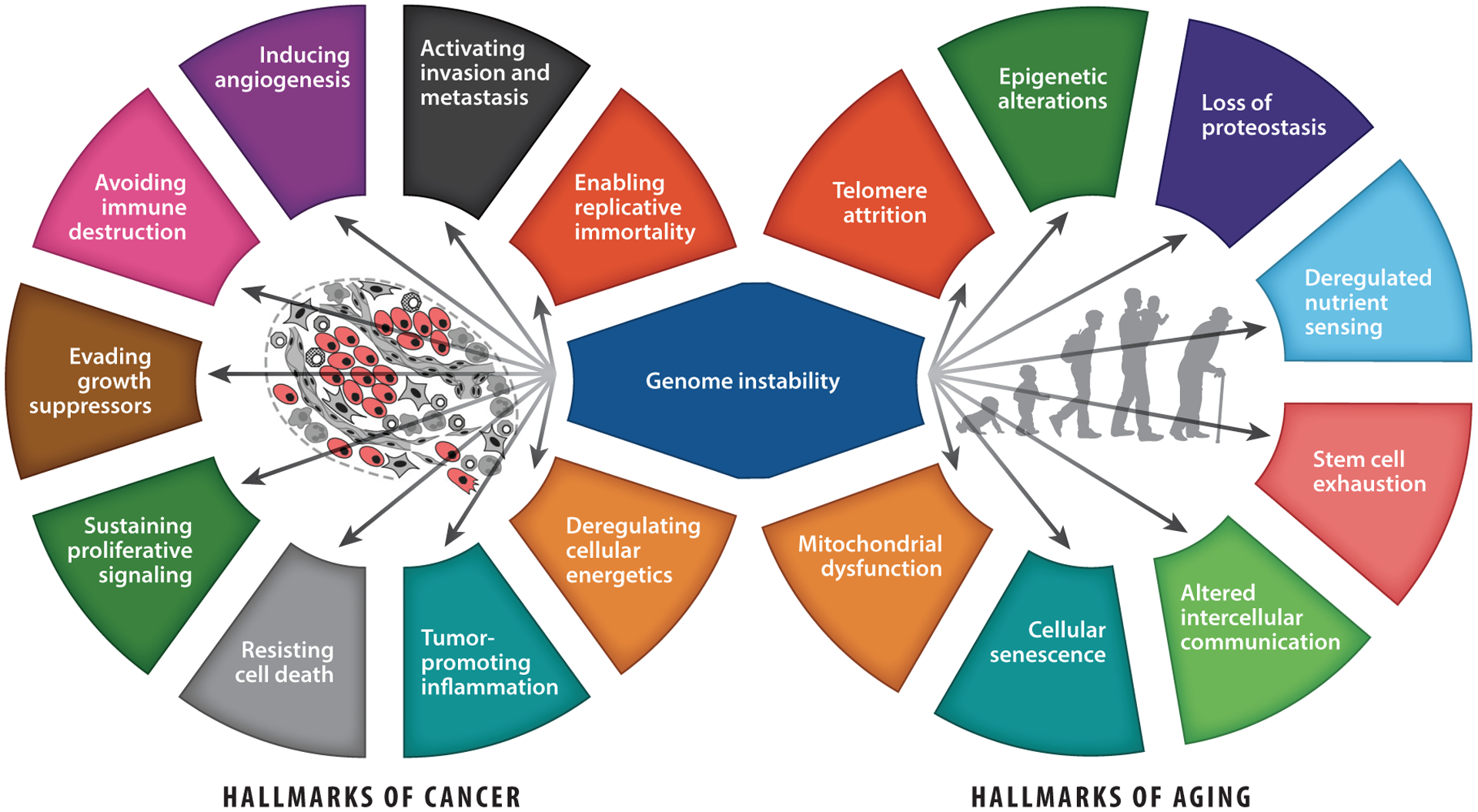
The hallmarks of cancer and aging intersect on genome instability. (*Left*) The ten hallmarks of cancer, proposed by Hanahan & Weinberg (2011), and (*right*) the nine hallmarks of aging, proposed by Lòpez-Otin et al. (2013), with genome instability as a common driving hallmark between both. Arrows indicate potential effects of genome instability on other hallmarks, and tumor cells are indicated in red. Center-left image adapted with permission from Hanahan & Weinberg (2011); copyright 2011 Elsevier.

Cancer and aging are in many respects each other’s counterpart, but they are also intimately interconnected. For both, a set of hallmarks has been identified, with genome instability as a common driving hallmark (Hanahan & Weinberg 2011, López-Otín et al. 2013) (Figure 1). Genome instability can be defined as the accumulation of genetic damage over time, such as structural changes in DNA and chromosomal aberrations. As our DNA is constantly being challenged by a variety of agents and spontaneous reactions, our cells rely on a proper DNA damage response and are equipped with an array of DNA repair processes (Bartek et al. 2007, Hoeijmakers 2001, Lindahl 1993). These DNA maintenance mechanisms are not 100% efficient, which leads to a slow, gradual accumulation of DNA damage with age. Upon DNA replication, damage can yield mutations and chromosomal aberrations, thereby driving tumorigenesis (Hanahan & Weinberg 2011, Hoeijmakers 2001, Negrini et al. 2010). Alternatively, persisting DNA damage can block replication, prompting cell cycle delay or arrest and cellular senescence, or it can trigger transcription stress, resulting in cellular functional decline and cell death, all of which contribute to aging and aging-associated diseases (Hoeijmakers 2009, Lans et al. 2019, López-Otín et al. 2013).

The importance of genome maintenance as a mechanism underlying both cancer and aging was further elucidated by the identification of human genotype-phenotype relationships, as mutations in genes implicated in DNA repair—leading to increased genome instability—enhance cancer susceptibility and/or accelerate aging (Hoeijmakers 2009, Marteijn et al. 2014, Niedernhofer et al. 2018, Vermeij et al. 2014).

Multiple signs of accelerated aging, such as cellular senescence, early graying, cachexia, osteoporosis, liver and kidney aging, and shortened lifespan, were shown for the first time in mice with defective DNA repair caused by mutations identified in rare progeroid diseases in humans (De Boer et al. 2002, Niedernhofer et al. 2006, Weeda et al. 1997). This breakthrough inferred a direct relationship between DNA damage exposure, DNA repair efficiency, and the rate of aging, consistent with the damage accumulation theory of aging (Kirkwood 2005), and it predicted that DNA-damaging chemotherapeutics used in cancer treatment may promote aging, which has now been confirmed in children and adults cured from cancer (Cupit-Link et al. 2017, Maccormick 2006).

Nutritional preconditioning (NP) can boost defense mechanisms and reduce DNA damage levels, which so far have been mostly studied in relation to aging. As genome instability is one of the driving hallmarks of aging and cancer (Figure 1), this review focuses on the relation between DNA damage and (accelerated) aging or cancer, as well as the use of NP as a counteractive measure.

## THE LINKS BETWEEN DNA DAMAGE, AGING, AND DIETARY RESTRICTION

DNA damage occurs in every cell at a rapid pace, originating from both endogenous (e.g., reactive metabolites) and exogenous (e.g., UV radiation, X-rays, genotoxins) sources. It is estimated that each mammalian cell experiences up to 10^5^ DNA lesions per day (Gates 2009, Lindahl & Barnes 2000, Swenberg et al. 2011, Tubbs & Nussenzweig 2017), ranging from spontaneous hydrolysis causing abasic (mainly apurinic) sites to deaminated bases, different types of single- and double-stranded breaks, DNA-DNA and DNA-protein cross-links, and about 100 types of oxidative DNA lesions (Cadet et al. 2002, Lindahl 1993, Nakamura & Swenberg 1999). Most of these lesions are removed by an intricate network of complementary DNA repair processes, including mechanisms that resolve damaged bases containing subtle alterations (e.g., oxidative lesions, alkylation damage) by BER (base-excision repair), bulky helix-distorting adducts throughout the genome by GG-NER (global genome nucleotide excision repair), lesions blocking ongoing transcription by TCR (transcription-coupled repair), double-stranded DNA breaks by NHEJ (nonhomologous end-joining) or by HR (homologous recombination), or interstrand DNA cross-links by cross-link repair pathways (Hoeijmakers 2001). Occasionally, however, the damage is unrepairable or not recognized or the repair is simply too late or error prone. This leads to a gradual increase of persisting DNA lesions over time, with genome instability as a consequence (Hoeijmakers 2001, Vijg 2014). Some lesions may occur in actively transcribed genes, causing transcription stress, which leads to altered gene expression (Lans et al. 2019), which in turn may indirectly influence several of the other important hallmarks of aging (Vermeij et al. 2016b) (Figure 1).

The effect of persisting DNA lesions on the process of aging is even more evident when genome integrity mechanisms (e.g., DNA repair systems) are affected. This is the case for a broad variety of rare progeroid syndromes, including Cockayne syndrome (CS), trichothiodystrophy, Werner syndrome, Nijmegen breakage syndrome, and Bloom syndrome, in which patients exhibit multiple features of premature aging (Vermeij et al. 2016b). Virtually all accelerated aging syndromes in humans exhibit this link with genome instability. For many of the genes affected, mouse models have been generated to better understand these progeroid disorders and the biomedical consequences of DNA damage (Carrero et al. 2016, Jaarsma et al. 2013). Several of these mouse models appear to be excellent paradigms of the human syndromes, in which several features were first identified in mice before being confirmed in patients (De Boer et al. 2002, Vermeulen et al. 2001). One such example is the *Ercc1*^Δ/−^ mouse model (Niedernhofer et al. 2006, Weeda et al. 1997), which is defective in multiple DNA repair pathways (Ahmad et al. 2008, Gillet & Schärer 2006, Kuraoka et al. 2000, Niedernhofer et al. 2004). Consequently, all cells and tissues accumulate many different types of DNA lesions faster than normal, leading to functional decline and widespread accelerated aging in postmitotic and proliferative organs and tissues. This limits their lifespan by 4–6 months, during which they progressively develop frailty and numerous age-related pathologies commonly observed in the elderly (Dollé et al. 2011, Vermeij et al. 2016b).

Besides premature aging, accumulation of DNA damage in the *Ercc1*^Δ/−^ and other DNA repair–deficient mouse models also triggers a protective response that alters energy metabolism, nutrient sensing, and redox status (Milanese et al. 2019, Niedernhofer et al. 2006). This antiaging survival response resembles dietary restriction (DR), involving the suppression of the GH (growth hormone)/IGF1 somato-, lacto-, and thyrotropic hormonal axes and upregulation of antioxidant defenses and resilience mechanisms, presumably in an attempt to extend lifespan by redirecting resources from growth to cellular maintenance and stress resistance (Niedernhofer et al. 2006, Schumacher et al. 2008, Van Der Pluijm et al. 2006). By limiting the investment of energy resources to organismal growth, this response also explains why progeroid DNA repair–deficient mice and patients remain so small (Laugel 2013). Strikingly, when exposed to agents inducing persistent transcription-blocking DNA lesions, wild-type (WT) mice and normal cells respond in a similar fashion, attenuating growth and supporting maintenance mechanisms, resembling the longevity response induced by DR (Garinis et al. 2009).

## CONSERVED RESPONSE OF REDUCED DIETARY INTAKE

The first scientific evidence that reducing food intake prolongs life was provided by two studies from the early 1900s. Osborne and colleagues noted that four rats that were given a restricted diet for various periods of time to retard their growth were among the longest lived in their study (Osborne et al. 1917). More systematically analyzed, a landmark paper for the field of DR (also often termed calorie restriction) was published by McCay and colleagues in 1935 that clearly demonstrated that restriction of food intake by 40% dramatically extended the lifespan of rats (McCay et al. 1935). Over the years, many more species have been examined, ranging from yeast and worms to flies and mice in various genetic backgrounds (Arslan-Ergul et al. 2016, Comfort 1963, Fontana et al. 2010, Goldman et al. 1999, Jönsson 2007, Kenyon 2010, Liao et al. 2010, Pifferi et al. 2018, Weindruch & Sohal 1997, Weindruch et al. 1986). These studies have highlighted the strong evolutionary conservation of the extension of lifespan by reduced dietary intake. For a while, researchers questioned whether this mechanism of extending lifespan only applies to model organisms under laboratory conditions or whether it could also be applied to humans.

The first clear indications arose from DR studies in nonhuman primates by Colman et al. (2009) at the University of Wisconsin (UW) and Mattison et al. (2012) at the National Institute of Aging (NIA). Both research groups used rhesus macaques as experimental animals, subjecting them to 30% DR. Effects on health span confirmed by both research groups include reduced incidence of diabetes and cancer and overall younger appearance of animals subjected to DR. The lifespan of the animals was increased significantly only in the study by Colman et al. (2009), where only 50% of control animals were still alive at the moment of analysis, as opposed to 80% of DR animals. Evaluation of the study protocols revealed important differences between the two studies that might explain why Mattison et al. (2012) did not find a clear effect on lifespan (Austad 2012, Colman et al. 2014, Mattison et al. 2017): The control animals at NIA did not receive bona fide ad libitum (AL) feeding but instead were given a slightly (~10%) restricted diet, reducing the difference with the 30% DR group (Colman et al. 2014, Mattison et al. 2017). Despite the different outcomes on survival, both UW and NIA studies showed improved health spans and thereby beneficial effects of DR in nonhuman primates.

## MECHANISTIC INSIGHTS FROM APPLYING DIETARY RESTRICTION TO DNA REPAIR–DEFICIENT MUTANTS

Despite decades of research, the mechanisms underlying life- and health span extension by DR are still poorly understood. These may involve increased stress resistance and resilience mechanisms; improved antioxidant defenses; alterations in GH, thyroid hormone (TH), and IGF1/insulin signaling; release of appetite-regulating hormones leptin and ghrelin; altered mitochondrial function, including greater utilization of lipids when compared with carbohydrates; activation of NAD^+^ metabolism and enhancement of mitochondrial redox regulation; reduced mammalian target of rapamycin (mTOR)-mediated translation; increased autophagic responses; induced sirtuin regulation; remodeling of fat tissue; and a shift from a pro- to an anti-inflammatory profile of circulating adipokines (Finkel 2015, Hoshino et al. 2018, Madeo et al. 2019, Speakman & Mitchell 2011). Among these mechanisms, the nutrient signaling pathways IGF1 and mTOR are evolutionary most conserved (Fontana et al. 2010), while others might be secondary consequences, dependent on cell type, or influenced by the biological clock, rather than affected by aging itself or, alternatively, regulated during feeding or fasting periods (Barzilai et al. 2012, De Cabo & Mattson 2019, Speakman & Mitchell 2011).

To gain a better understanding of these causes and consequences and to identify essential pathways for promoting longevity through diet, researchers have used model organisms harboring genetic defects. Experiments using *Caenorhabditis elegans* have highlighted the importance of nutrient-sensing mechanisms and showed the effector genes *daf-16*/FoxO and *aak-2*/AMPK to be essential for mediating the longevity effect induced by DR (Greer & Brunet 2009, Mair & Dillin 2008). Applying DR to DNA repair–deficient mice was expected to yield similar outcomes to AL-fed littermates, as they already have an activated DR-like antiaging survival response (Garinis et al. 2009, Schumacher et al. 2008). Unexpectedly, the *Ercc1*^Δ/−^ progeroid mice responded to an extraordinary degree to the application of DR: Median and maximum remaining lifespans were extended by approximately 200% in both genders (Vermeij et al. 2016a). Similar results were obtained in DNA repair–deficient progeroid *Xpg*^−*/*−^ mice, a model for a severe form of CS combined with the human repair syndrome xeroderma pigmentosum (Barnhoorn et al. 2014, Vermeij et al. 2016a). DR drastically improved health span, retarding all aspects of premature aging examined, including hepato-, nephro-, immuno-, osteo-, and vascular aging, but most notably neurodegeneration: DR preserved 50% more neurons and fully prevented motoric dysfunction, which has major clinical implications. Importantly, this study showed that the already activated survival response was further enhanced by DR, and it identified reduced DNA damage levels and attenuated transcription stress as novel effects of DR that can explain the exaggerated response of DNA repair–deficient mice to DR (Vermeij et al. 2016a).

In the case of laboratory animals, the origin of most of the damage must be endogenous and—in view of the notion that DR has such a strong effect—must be influenced by the amount of food. If food were the sole source of the damage, however, it would be difficult to explain why 30% restriction would exert such a dramatic effect. Therefore, DR must trigger an active program that can delay aging and reduce genome damage. Although improving DNA repair would be an option for reducing genomic injuries, this is unlikely in the case of the *Ercc1*^Δ/−^ and *Xpg*^−/−^ mice, as multiple repair systems are genetically inactivated in these mutants and effective compensatory pathways are unknown. This suggests that not (or not solely) enhanced damage removal but reduced damage induction and perhaps altered damage responses are the focuses of DR. Indeed, reducing glucose intake and that of other nutritional components leads to a redesign of major metabolic routes (such as glycolysis, oxidative phosphorylation, and pentose phosphate shunt) and alters mitochondrial function. This lowers the respiratory exchange ratio and production of reactive oxygen species (ROS) that could normally damage DNA, but also enhances mitochondrial reducing equivalents that quench ROS. Moreover, it results in a lower body temperature, reducing thermodynamics and decreasing ROS damage to macromolecules (Conti et al. 2006, Finkel 2015, López-Otín et al. 2016, Speakman & Mitchell 2011). Other energy intermediates are directly interacting with nutrient-sensing pathways such as amino acids with mTOR, AMP/ADP with AMPK, NAD^+^/NADH with sirtuins, and fatty acids with PPARs (peroxisome proliferator-activated receptors) (Balasubramanian et al. 2017, Finkel 2015). DR reduces circulating levels of IGF1, insulin, GH, and TH, thereby temporarily suppressing growth, and boosts antioxidant capacities and stress-resistance mechanisms (Bartke 2019, Gillespie et al. 2016), again dampening the induction and negative consequences of DNA damage.

Many of these mechanisms still pop up in DR expression profiles of *Ercc1*^Δ/−^ mice that actually had this response already activated under AL conditions in response to accumulated DNA damage levels. Surprisingly, *Ercc1*^Δ/−^ and WT littermates on DR revealed a very similar consistent response: 684 genes out of the 688 common genes upon DR responded concordantly between *Ercc1*^Δ/−^ and WT mice, including the GH/IGF1 axis and antioxidants (Vermeij et al. 2016a). Therefore, reducing the generation and enhancing the elimination of metabolic byproducts such as ROS that otherwise would wreck the cell’s interior, including nuclear and mitochondrial DNA, are likely prime targets of DR.

## CHEMOTHERAPY ACCELERATES AGING

The observation that DNA damage is the main driver of aging implies that other instances where DNA damage occurs can benefit from NP. It has become clear that DNA damage–inducing anticancer therapies accelerate aging, most clearly in childhood cancer survivors (Hudson et al. 2013, Maccormick 2006). Since the survival of cancer patients has increased over the last decades, maintaining quality of life is becoming increasingly important. Cancer survivors have, later in life, a higher risk for many pathologies, including frailty, cardiovascular disease, hypertension, stroke, secondary neoplasms, cataracts, low bone mineral density, metabolic syndrome, diabetes, primary hypogonadism, and cognitive decline (Ness et al. 2018), and they develop these at a younger age compared to healthy individuals (Bhakta et al. 2017, Cupit-Link et al. 2017). The clinical phenotype termed frailty (a combination of decreased lean muscle mass, decreased vitality, poor physical activity, and slowness or weakness) in adult childhood cancer survivors reveals an aging tendency comparable with that in the elderly, already two decades earlier than expected (Hayek et al. 2020, Ness & Wogksch 2020, Ness et al. 2017, Smitherman et al. 2018). As with aging, DNA damage is the primary cause of side effects in cancer patients due to intense toxic treatment regimens, which opens possibilities for the use of dietary interventions in cancer treatment (see the sidebar titled Types of Nutritional Preconditioning).

## APPLICATIONS OF NUTRITIONAL PRECONDITIONING IN CLINICAL CANCER TREATMENT

NP has the potential to improve quality of life and therapy of cancer patients by preventing short-term toxicities, improving therapeutic efficacy, and reducing late-life effects. DNA-damaging treatments including radio- and most chemotherapies cause local or systemic acute genotoxicity and hence local or systemic accelerated cell death, functional decline, and, therefore, aging. Nutritional strategies have been shown to reduce endogenous DNA damage levels and to boost maintenance and resilience mechanisms (Cabelof et al. 2003, Morselli et al. 2010, Solanas et al. 2017, Vermeij et al. 2016a), which may protect the body from exogenous damages (e.g., chemotherapy) as well. By inducing such a protective survival response prior to DNA-damaging treatments, NP might prevent (part of) these toxicities at least in normal tissues, and probably to a lesser extent or not at all in the tumor, thereby alleviating short- and long-term side effects and the burden experienced by cancer patients. Moreover, NP can be important for other aspects of cancer treatment. In the following sections, different applications relevant for the well-being of cancer patients are discussed.

### Surgery

NP is highly relevant for surgery. Surgical removal of tumors is an important part of treatment, especially for patients with solid tumors. The blood flow to the tissue surrounding the tumor is temporarily restricted and restored at the end of operation. The temporary lack of oxygen and other nutrients (ischemia), followed by reperfusion, is associated with massive formation of ROS, causing acute tissue damage including oxidative DNA lesions, enhancing cell death and inflammation (Kalogeris et al. 2012), and thereby actually causing local aging. As demonstrated below, both DR and fasting strongly protect against ischemia reperfusion injury (IRI), both in model organisms and patients.

For example, mice subjected to bilateral kidney clamping to induce renal IRI that were preconditioned by 30% DR for 2 or 4 weeks or 2 or 3 days of fasting all survived the surgical procedure and retained superior renal function in comparison to their AL-fed littermates, of which only 40% survived (Mitchell et al. 2010).

Fasting was also shown to reduce damage to the brain after stroke. Sprague-Dawley rats that were subject to a fasting diet for 3 days before receiving cerebral artery occlusion showed significant neuroprotection (Varendi et al. 2014).

Preoperative fasting or DR studied in animal models has also been shown to be beneficial in IRI settings related to, among others, the heart (Shinmura et al. 2005, Wan et al. 2010), liver (Menezes-Filho et al. 2017, Verweij et al. 2011), retina (Kawai et al. 2001), and revascularization (Kondo et al. 2009), indicating the systemic nature of this response.

Clinical trials looking into the effects of preoperative NP are scarce but show promising results. Van Nieuwenhove et al. (2011) studied patients scheduled for laparoscopic gastric bypass surgery in a multicenter, randomized, single-blind study. In total, 273 patients were followed, of which 137 followed a very low-calorie diet (VLCD) for 2 weeks. The main finding of the study was the reduction of postoperative complications for the VLCD group, most clearly for wound infections (Van Nieuwenhove et al. 2011).

Beneficial effects were also shown in patients—of which 94% underwent cancer resection—undergoing liver surgery (Reeves et al. 2013). These patients have a higher risk of complications when having steatosis or steatohepatitis (McCormack et al. 2007, Vauthey et al. 2006). Both pathologies were significantly lowered in patients that performed DR 1 week before surgery, with a caloric intake of 900 kcal/day. Additionally, there was a trend for reduced severity in steatosis and significantly less blood loss in the DR group (Reeves et al. 2013).

### Short- and Long-Term Toxicity

The list of side effects caused by radiotherapy and various types of chemotherapy is a very long one. Problems arising during and shortly after therapy hamper normal functioning of patients, affecting quality of life and interfering with therapy regimen, and require ways to reduce suffering without diminishing treatment efficacy. In contrast to many tumor cells, normal cells adapt their metabolism upon nutritional intervention, enhancing stress resistance, making the cells less responsive to harmful agents, which is sometimes termed differential stress resistance (DSR) (Raffaghello et al. 2008).

As most DNA-damaging chemotherapeutics are administered intravenously, toxicity can occur throughout the body. Toxicities induced by DNA damage vary depending on the type of damage (pharmacokinetics and -dynamics of the chemotherapeutic compounds) and the cellular context (such as the proliferation rate, repair capacity, vascularization, etc. of tissues). NP-induced protection against various classes of chemotherapy—i.e., induction of DSR—has been shown using different in vitro and in vivo models.

Doxorubicin, a topoisomerase II inhibitor that blocks the unwinding of DNA (Tewey et al. 1984), thereby inhibiting DNA replication, causes strand breaks. It is also implicated in free radical formation and histone deregulation (Feinstein et al. 1993, Pang et al. 2013). Doxorubicin’s main side effect is cardiotoxicity (Lefrak et al. 1973).

Following 48 hours of food deprivation, doxorubicin-treated CD-1 mice showed 100% survival, in contrast to 38% of mice that received Igf1 injections every 12 hours during the fasting period (Lee et al. 2010).

Liver-specific deletion of *Igf1r* leads to a reduction of 80% of circulating Igf1 levels in mice (LID mice) (Lee et al. 2010), similar to levels established by DR, and can be used to mimic NP. Of the non-tumor-bearing mice, only 25% of control (WT) mice survived two cycles of doxorubicin treatment, whereas all LID mice survived. In melanoma-bearing xenograft mice, 60% of LID mice were still alive 90 days after doxorubicin treatment, whereas none of the treated WT mice survived (Lee et al. 2010). Unfortunately, the authors did not include nontreated controls, making it impossible to determine the effect of only lowered circulating Igf1 levels on survival.

Another topoisomerase II inhibitor, etoposide, prevents religation of DNA strands, thereby causing DNA breaks (Robinson & Osheroff 1991). High-dose treatment was tested in A/J, CD-1, and Nude-nu mice, which were fasted for 2–3 days and subsequently treated with equitoxic doses. In all three models, signs of toxicity, including ruffled hair, kyphosis, and decreased locomotor activity, were clearly present in AL-fed animals but were mild or absent in fasted animals. Etoposide treatment led to weight loss in the first days after treatment, whereas fasted animals gained weight. Survival increased tremendously in fasted mice, as only one mouse from the prefasted group (*n* = 28) died shortly after treatment from all three backgrounds. From the control mice, 43%, 100%, and 56% had died at 10, 5, and 5 days after treatment for the A/J, CD-1, and Nude-nu groups, respectively (Raffaghello et al. 2008).

Besides toxicity caused by a specific drug, toxicity can be elevated due to interactions between multiple drug treatments. Survival of C57BL/6 mice treated with doxorubicin was significantly lower for mice that received multiple injections of dexamethasone (normally added to treatment to lower nausea and vomiting) before doxorubicin treatment. Two-day fasting before doxorubicin treatment reversed this effect and even increased survival compared to control animals, which were only treated with doxorubicin. At 20 days after treatment, this resulted in 5%, 90%, and 100% survival for animals that received dexamethasone, dexamethasone and fasting, or fasting alone alongside doxorubicin treatment, respectively, whereas doxorubicin treatment alone led to 25% survival. Signs of cardiotoxicity were diminished in animals that were fasted before doxorubicin injection compared to AL-fed animals. No data on cardiotoxicity were available for mice additionally treated with dexamethasone. Dexamethasone causes hyperglycemia, which was shown to be (partially) causal for the increased toxicity, as the addition of insulin alongside dexamethasone (restoration of euglycemia) leads to reduced toxicity; glucose injections before fasting (leading to hyperglycemia) resulted in increased toxicity. Additional experiments showed that fasting leads to increased local concentrations of atrial natriuretic peptides, B-type natriuretic peptides, and cardioprotective peptides, which are probably implicated in reduced cardiotoxicity in fasted animals after doxorubicin treatment (Di Biase et al. 2017).

As a last example, cisplatin is a chemotherapeutic that forms intra- and interstrand cross-links with the DNA, thereby preventing transcription and replication (Siddik 2003). A significant reduction in cisplatin-induced nephrotoxicity was observed in mice that were fasted for 3 days before cisplatin treatment. Histological parameters including tubule damage, glomerular deterioration, brush border degradation, and serum measurements including creatinine and urea levels all indicated less severe kidney damage in fasted mice compared to AL-fed mice (Gunebakan et al. 2020). Together, these findings indicate that NP can induce protection in healthy tissue against multiple types of DNA-damaging chemotherapeutics.

Lastly, NP can be used to reduce long-term toxicity. Especially for childhood cancer survivors, this benefit may be very significant in view of their longer remaining life expectancy and the fact that they experience aging-like pathologies earlier in life compared to the normal population (Armstrong et al. 2014, Hudson et al. 2015, Schuitema et al. 2013). Apart from survival outcomes discussed above, no studies have yet been performed to specifically look at the effects of NP on long-term toxicity, but evidence in the field of aging research supports its potential for delaying aging in general and reduction of DNA damage–driven accelerated aging (Barnhoorn et al. 2014, Vermeij et al. 2016a). Furthermore, the implementation of an NP for acute toxicity might also lead to less accumulation of DNA damage during treatment and therefore prevent or reduce pathologies later in life.

### Tumor Growth

Besides preventing toxicities to healthy tissues and organs, NP can also impact the tumor itself. Cancer cells harbor mutations that lead to differentiation arrest, as well as uncontrolled growth due to constitutive activation of proliferative pathways or repression of antigrowth signaling. When the availability of nutrients is reduced, like in NP, many cancer cells cannot easily adapt—in contrast to normal cells—which may render them more vulnerable to damaging agents, a condition termed differential stress sensitization (DSS) (Lee et al. 2012).

This difference in responses between tumor cells that have difficulties in adapting to NP conditions and normal cells that go into defense mode can already result in tumor shrinkage by the NP regimen per se. In vitro, the majority of 17 human and murine cancer cell lines showed decreased survival when starved and treated with doxorubicin or cyclophosphamide compared to either chemotherapeutic alone (Lee et al. 2012). In vivo mouse experiments on various allograft or xenograft models for breast cancer, melanoma, glioma, and ovarian cancer showed reduced tumor growth or tumor shrinkage after 2 days of fasting compared to an AL diet, which was further reduced in combination with chemotherapy (Lee et al. 2012). The effect of fasting on tumor growth was also tested in multiple in vivo metastatic tumor models, including breast cancer, melanoma, and neuroblastoma models. In all cases, mice that were fasted and received chemotherapy showed the longest survival (Lee et al. 2012). Strikingly, even the number of metastatic sites was reduced in several of the tumor models tested.

Similar differential effects of DR on solid tumor growth were found by Kalaany & Sabatini (2009). Six human tumor lines were injected subcutaneously into NOD/SCID (nonobese diabetic/severe combined immunodeficiency) mice, representing prostate, colon, brain, and three different breast cancers. After 2–3 weeks of AL feeding or 40% DR, the colon cancer and two breast cancer tumors were significantly hampered in growth in the DR mice, whereas the prostate cancer, brain cancer, and one of the breast cancer tumors grew to a similar size under AL and DR conditions. As all restricted mice lost equal amounts of body weight and showed a similar decrease in blood plasma Igf1 and insulin levels, the authors hypothesized that the differential response of tumor types should be attributed to a tumor-intrinsic factor. The DR-resistant tumors had aberrant PI3K signaling, either via PTEN loss or mutated PI3K, leading to constitutive PI3K expression. During DR, increased expression of PTEN was required to attenuate tumor volume, as induced expression of PTEN in a PTEN-null model reversed DR resistance. Reduction of tumor size was due to either decreased proliferation or increased apoptosis (Kalaany & Sabatini 2009).

The DSS induced by fasting also applies to hematological cancer types. Mouse models for B cell acute lymphoblastic leukemia (B-ALL) were subjected to four fasting cycles without chemotherapy treatment, alternating between 1–2 days of fasting and 1–2 days of refeeding (Lu et al. 2017). Fasting cycles were performed either 2 days after tumor cell transplantation or at a later time point when a proper tumor was established. Fasting shortly after initiation of B-ALL led to a significant increase in survival, where 75% of fasted animals were still alive at the end of the experiment, at least 61 days longer than fed animals. Survival for animals that were fasted after tumor establishment was at least 62 days longer for 60% of the fasted mice than fed controls. Similar results were obtained for T cell acute lymphoblastic leukemia (T-ALL). Four cycles of fasting after T-ALL induction resulted in 40% survival until the end of the experiment, at least 30 days longer than control animals, of which none survived. When T-ALL was already established, 50% of fasted animals survived for at least 68 days longer than control animals. The authors did not find such an effect of fasting-only on acute myeloid leukemia (AML). The difference in response between ALL and AML was attributed to leptin receptor (LEPR) expression, which was increased after fasting in both B-ALL and T-ALL cells, but was already high in AML and did not increase over time. Overexpression of LEPR drives the differentiation of B-ALL tumor cells into terminal B cells, which are nonmalignant (Lu et al. 2017).

Interestingly, LEPR expression is upstream of PI3K and could interfere with PI3K signaling, consistent with the importance of this pathway in response to DR and fasting, although this does not rule out the relevance of other molecular mechanisms. Due to specific mutations present in different tumor types, the response to NP might depend on the tumor type and mutation (Kanarek et al. 2020). However, independent of tumor status, NP might still have the benefit of reducing therapy-induced side effects.

## IMPLEMENTATION OF NUTRITIONAL PRECONDITIONING INTO CANCER TREATMENT SCHEDULES

**Figure 2:**
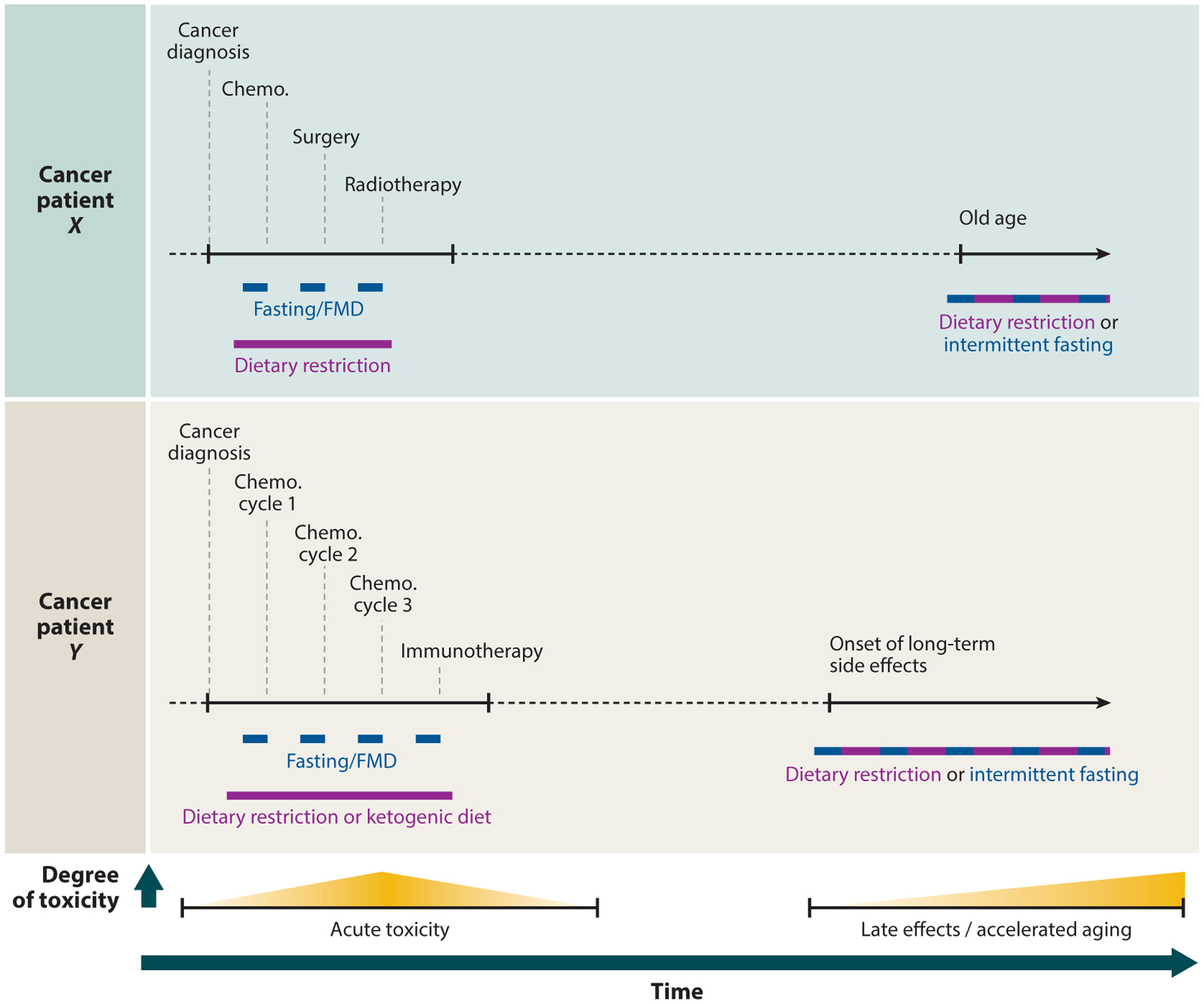
Conceptual overview of nutritional preconditioning implemented alongside cancer treatment (Table 1, Supplemental Table 1) or at older age to prevent side effects of cancer treatment or aging (Supplemental Table 2). Dark blue bars indicate periods of 1–5 days, and purple bars indicate periods of multiple weeks. Abbreviations: chemo., chemotherapy; FMD, fasting-mimicking diet.

The health effects of NP alongside standard-of-care cancer therapy are currently being investigated in several clinical trials (Table 1, Supplemental Table 1). The start time, duration, type, and degree of the intervention depend on the specific situation and application and should be compatible with additional therapy (Figure 2). Several additional considerations need to be considered to assure the safety of the patient. First, the physical condition of the patient must be adequate to tolerate a nutritional intervention. Patients that are suitable must be closely monitored and guided by health-care professionals during the whole intervention for parameters such as body weight, food intake, well-being, and physical status. As discussed below, only a few trials have been completed so far, and most evidence for favorable effects of NP has been obtained from mouse studies. More studies with human subjects are required before NP (Figure 2) can become standard practice. The exact schedule and composition of a dietary intervention should be tailored to the treatment schedule and to the specific tumor type to ensure that the survival response is maximally established at the moment damage is occurring and does not enhance tumor growth or toxicity.

## CLINICAL TRIALS IMPLEMENTING NUTRITIONAL PRECONDITIONING AGAINST CANCER IN HUMANS

The number of trials in which cancer patients undergo any form of NP is limited but has been increasing especially in the last decade (Table 1, Supplemental Table 1). These trials combine different types of NP with chemotherapy, radiotherapy, or surgery in a range of cancer types. The first few trials have been completed, and they confirm the feasibility of implementing adapted feeding strategies into cancer therapy and the safety of patients undergoing NP (Bauersfeld et al. 2018, De Groot et al. 2015, Dorff et al. 2016, Safdie et al. 2009).

Most studies have applied some form of short-term fasting (STF), ranging from 48 to 180 hours. All studies confirm the feasibility and safety of STF alongside chemotherapy. In some cases side effects of fasting were noted, such as dizziness, nausea, and headache, but were considered to be minor (Bauersfeld et al. 2018, Safdie et al. 2009). Overall, the studies showed (a trend towards) reduced side effects attributable to chemotherapy in patients that underwent fasting compared to nonfasted patients. In some studies (De Groot et al. 2015, Dorff et al. 2016), plasma measurements were performed to assess several key metabolic markers, including glucose, insulin, and IGF1. Only IGF1 showed a significant decrease upon fasting (De Groot et al. 2015). Both research groups investigated DNA breaks in peripheral blood mononuclear cells (PBMCs) by either comet assay (Dorff et al. 2016) or γH2AX intensity (De Groot et al. 2015) and revealed lower DNA damage induction following chemotherapy or a faster recovery of PBMCs in fasted patients.

Another diet frequently assessed is the ketogenic diet (KD), mostly studied in patients suffering from brain cancer, but available data are very limited. Besides gliomas, completed trials have also assessed nonbrain tumors (Cohen et al. 2018a,b; Klement & Sweeney 2016; Klement et al. 2019; Martin-McGill et al. 2020; Rieger et al. 2014; Tan-Shalaby et al. 2016; Zahra et al. 2017). Unfortunately, these studies mostly included only a small number of patients or, when started with a larger cohort, experienced dropout during the study (mostly due to scheduling conflicts or other non-diet-related reasons). The main focus of most trials has included the safety and feasibility of KD, as well as the effect of KD on quality of life in cancer patients. The consensus seems to be that KD is feasible and safe in patients with different types of cancer. The combined results suggest that a KD for a maximum of 5–6 weeks consisting of a ratio of fat to other macronutrients of less than 4:1 is most promising as a feasible intervention.

Patients that have been able to stay on a KD mostly reported no severe adverse effects due to the diet and reported that their quality of life was stable or improved (Cohen et al. 2018a, Martin-McGill et al. 2020, Tan-Shalaby et al. 2016). Measurements on body composition have indicated a positive alteration by KD, with a decrease in fat mass and an increase in fat-free mass and skeletal muscle mass (Cohen et al. 2018a, Klement et al. 2019). Taken together, these results might suggest that KD leads to a more beneficial body composition. Use of KD might lead to better treatment response in overweight patients, as it is reported that patients with obesity or low muscle mass might have a lower response to treatment (Calle et al. 2003, Malietzis et al. 2016, Prado et al. 2009, Yip et al. 2015).

An alternative to complete STF is a fasting-mimicking diet (FMD), whose effect has been studied in a few clinical trials. The one FMD trial with published results so far applied FMD to HER2-negative breast cancer patients receiving chemotherapy (De Groot et al. 2019). Unfortunately, the study was terminated due to low compliance, as some patients reported a dislike of the taste of the diet and developed an aversion to food close to chemotherapy treatment. Some of the patients decided to fast instead of undergoing an FMD for part of their chemotherapy cycles or even their whole treatment schedule. Analysis of the FMD group, irrespective of compliance, still showed indications of decreased DNA damage after chemotherapy in lymphocytes compared to the DNA damage levels in the control group (De Groot et al. 2019). Hence, NP appears to improve health parameters and to reduce side effects of chemotherapy treatment.

## CONCLUDING REMARKS AND PERSPECTIVES

Although important benefits of DR have already been known for over a century, its potential has still not been fully determined. The relatively recent introduction of NP in cancer treatment already shows promising beneficial effects in different treatment areas (Table 1). Further clinical results await, but preliminary findings hold significant promise for patients. In addition to reduction of tumor volume and treatment-induced side effects, NP might also be of benefit for patients receiving radiotherapy, where DNA damage is inflicted to the healthy tumor environment as well; for patients receiving stem cell transplantation; or in the context of immunotherapy, as numerous immunological parameters also change with DR or fasting.

As the survival response can also be used to reduce or slow down aging-related pathologies, introduction of NP in many more contexts, such as research related to dementia or cardiovascular or autoimmune diseases, could reveal a whole new modality for treatment or prevention (Supplemental Table 2). Of course, in-depth research in these and other areas has to be performed to test this hypothesis. When NP is used, the safety and general health of the patient are of utmost concern, which requires comprehensive analysis of the patient’s physical status before starting NP and careful monitoring during NP. When adapted to many characteristics such as the patient’s health status, age, activity level, disease, and treatment schedule, NP has the potential to decrease the burden and improve the quality of life for patients at very low costs.

## Supplementary Material

Supplemental Material

## Figures and Tables

**Table 1:** Completed clinical trials studying the implementation of nutritional preconditioning in cancer treatment, including chemotherapy, surgery, and radiotherapy

Cancer type	Nutritional intervention	Effect on	Study focus	Study ID^a^	Location
Advanced or metastatic cancer^b^	KD	Tumor growth	Safety and tolerability of KD in advanced cancer patients	NCT01716468	Pennsylvania, USA, 1 center
Breast cancer	Low-fat diet	Tumor growth	Effect on disease-free and overall survival	NCT00002564	USA, 37 centers
Breast cancer	DR	Tumor growth (acute toxicity)	Feasability of DR and effect on tumor progression following surgery and radiotherapy	NCT01819233	Pennsylvania, USA, 1 center
Breast cancer	FMD	Acute toxicity, tumor growth	Grade III/IV toxicity of neoadjuvant chemotherapy	NCT02126449	The Netherlands, 15 centers
Breast cancer^b^	Fasting	Acute toxicity	Effect of STF on tolerance to adjuvant chemotherapy in breast cancer patients	NCT01304251	The Netherlands, 1 center
Glioblastoma^b^	KD	Tumor growth	Feasibility of KD	NCT00575146	Germany, 2 centers
Glioblastoma	KD/fasting	Tumor growth	Progression-free survival rates 6 months after reirradiation	NCT01754350	Germany, 2 centers
Glioblastoma^b^	KD	Acute toxicity	Adherence to KD	NCT03075514	United Kingdom, 1 center
Gynecological cancer^b^	Fasting	Acute toxicity	QoL after chemotherapy treatment	NCT01954836	Germany, 1 center
Malignant neoplasm	Fasting	Acute toxicity	Safety and feasibility of STF	NCT01175837	Minnesota, USA, 1 center
Ovarian cancer, endometrial cancer^b^	KD	NA	Changes in fasting glucose, fasting insulin, and beta-hydroxybutyrate; effect on body composition	NCT03171506	Alabama, USA, 1 center

Abbreviations: DR, dietary restriction; FMD, fasting-mimicking diet; KD, ketogenic diet; NA, not available; QoL, quality of life; STF, short-term fasting.

a Study ID refers to clinical trials registered at https://clinicaltrials.gov.

b Trials referred to in main text.

## TYPES OF NUTRITIONAL PRECONDITIONING

Over the years, nutritional preconditioning (NP) has been used in aging and cancer research. For the sake of clarity, the interventions frequently referred to in this review are here briefly explained.

Dietary restriction (DR) or caloric restriction (CR) refers to reduced intake without malnutrition. Nutritional intake is allowed during the day, but total caloric intake is generally reduced by 20–40%. More specific forms of DR exist in which the balance of macronutrients is affected or lowered.

The ketogenic diet (KD) has a high fat content, approximately 70%, primarily saturated, and is (very) low in carbohydrate content, but it is usually a normocaloric diet.

Short-term fasting (STF) means no food intake, only water, for a given period, in the range of days. This type of fasting is implemented in intermittent fasting, where periods of normal consumption are alternated with fasting.

Fasting-mimicking diet (FMD) is a diet lower in caloric intake than DR but for a longer period than STF. The most common FMD consists of a 5-day schedule of a plant-based low-calorie and low-protein diet, containing ~ 1,100 kcal on the first day, which is lowered to ~700 kcal on the subsequent 4 days (Wei et al. 2017).
