# Supplementary material for: Nutritional Preconditioning in Cancer Treatment in Relation to DNA Damage and Aging
Source: Annu Rev Cancer Biol. Author manuscript; Available in PMC 2022 Apr 25. (PMC9037985; doi:10.1146/annurev-cancerbio-060820-090737)
Supplement: Supplemental Material [file NIHMS1748750-supplement-Supplemental_Material.pdf]

Supplemental Material: Cancer Biol. 2021. 5.
https://doi.org/10.1146/annurev-cancerbio-060820-090737
Nutritional Preconditioning in Cancer Treatment in Relation to DNA Damage and Aging
van den Boogaard et al.

Supplemental table 1 – Clinical trials studying implementation of nutritional preconditioning in cancer treatment being chemotherapy, surgery or radiotherapy. DR, dietary restriction; FMD, fasting-mimicking diet; KD, ketogenic diet; QoL, quality of life; STF, short-term fasting; NA, not available. *Trials referred to in main text.

Cancer type	Nutritional intervention	Effect on	Study focus	Patient group	**Study name,** description	Study ID[a]	Location	Status
Advanced or metastatic cancer	KD	Tumor growth	Safety and tolerability of KD in advanced cancer patients	Male and female adults	**Ketogenic Diet in Advanced Cancer**	NCT01716468	Pennsylvania, USA, 1 center	Completed*
Advanced LKB1-inactive lung adenocarcinoma	FMD +- metformin	Tumor growth	Effect on progression-free survival	Male and female adults	**Metformin Plus/Minus Fasting Mimicking Diet to Target the Metabolic Vulnerabilities of LKB1-inactive Lung Adenocarcinoma (FAME)**	NCT03709147	Italy, 1 center	Not yet recruiting
Brain tumour	Ketogenic diet	Acute toxicity, Tumor growth	Feasibility, tolerability and tumour response	Boys and girls up to 21 yrs	**Therapeutic Targeting of Sex Differences in Pediatric Brain Tumor Glycolysis**	NCT03591861	Missouri, USA, 1 center	Recruiting
Breast cancer	Low-carb diet	NA	Effect on aromatase inhibitor therapy	Adult females	**BREADS: Breast Adjuvant Diet Study**: <20 g carbohydrates/day, 6 months	NCT04189263	California, USA, 1 center	Recruiting
Breast cancer	Low-fat diet	Tumor growth	Effect on disease-free and overall survival	Postmenopausal females	**Nutrition Intervention in Treating Women With Breast Cancer**: Total fat intake <15% of calories	NCT00002564	USA, 37 centers	Completed

Breast cancer	Low-carb diet	Tumor growth	Effect on preoperative endocrine prognostic index (PEPI)	Adult females	**NEOBREADS: Neoadjuvant Breast Diet Study (NEOBREADS)**: <20 g carbohydrates/day, 6 months	NCT03822715	California, USA, 1 center	Recruiting
Breast cancer	DR	Tumor growth (Acute toxicity)	Evaluate feasibility of DR and effect on tumour progression	Adult females	**Caloric Restriction in Treating Patients With Stage 0-I Breast Cancer Undergoing Surgery and Radiation Therapy (CAREFOR)**: 25% CR	NCT01819233	Pennsylvania, USA, 1 center	Completed, results available
Breast cancer	Low carb (ketogenic) diet, low fat diet	Tumor growth	Evaluation of proliferation and apoptosis markers	Adult females	**Comparison of Healthy Diets on Breast Cancer Markers (KetoBreast)**	NCT02744079	New York, USA, 1 center	Recruiting
Breast cancer	FMD	Acute toxicity, Tumor growth	Grade III/IV toxicity	Female adults	**DIetary REstriction as an Adjunct to Neoadjuvant ChemoTherapy for HER2 Negative Breast Cancer (DIRECT)**	NCT02126449	The Netherlands, 15 centers	Completed
Breast cancer (HER2-positive)	Fasting	Acute toxicity, Tumor growth	Safety of fasting, decrease in chemo-induced side effects, tumor shrinkage	Female adults	**Fasting on Newly Diagnosed Breast Cancer (STEFNE)**: fasting 24 h before and 24 h after chemotherapy	NCT02379585	Arizona, USA, 1 center	Terminated
Breast cancer	Fasting	Acute toxicity	Effect of STF on tolerance to adjuvant chemotherapy in	Female adults	**Effects of Short-term Fasting on Tolerance to Chemotherapy**: 24 h	NCT01304251	The Netherlands, 1 center	Completed*

			breast cancer patients		before and 24 h after chemotherapy			
Breast cancer	FMD +- metformin	Tumor growth (Acute toxicity)	Effect on treatment response	Female adults	**Calorie Restriction With or Without Metformin in Triple Negative Breast Cancer (BREAKFAST)**: every three weeks classical FMD	NCT04248998	Italy, 1 center	Not yet recruiting
Breast cancer, ovarian cancer	Fasting + vegan diet vs. vegan diet	Acute toxicity, Tumor growth	Effect on Functional Assessment of Cancer Therapy - General (FACT-G) score	Adult females	**Intermittent Fasting Accompanying Chemotherapy in Gynecological Cancers (FIT2):** Modified fasting 36-48h before and 24h after chemo 350-400 kcal per day. Between chemotherapies time-restricted feeding	NCT03162289	Germany, 8 centers	Recruiting
Breast cancer	DR	Acute toxicity	Effect on heart function (anthracycline treatment)	Adult females	**Caloric Restriction and Exercise for Protection From Anthracycline Toxic Effects (CREATE)**: 50% CR for 48h. Also includes exercise arm	NCT03131024	Alberta, Canada, 1 center	Recruiting
Breast cancer, colorectal cancer	FMD	Tumor growth	Safety and feasibility of FMD	Male and female adults	**Fasting-mimicking Diet in Patients Undergoing Active Cancer Treatment**	NCT03595540	Italy, 1 center	Recruiting

Breast cancer, melanoma	FMD	Tumor growth	Analysis of immunological and metabolic changes induced by FMD in pre- and post-operative setting	Male and female adults	**Impact of Dietary Intervention on Tumor Immunity: the DigesT Trial (DIgesT)**	NCT03454282	Italy, 1 center	Recruiting
Breast and prostate cancer	DR	Acute toxicity, Tumor growth	Effect on toxicity and efficacy of chemotherapy	Male and female adults	**Controlled Low Calorie Diet in Reducing Side Effects and Increasing Response to Chemotherapy in Patients With Breast or Prostate Cancer**: 3 d prior to chemo, 12 wks during chemo, 2 d after chemo	NCT01802346	California & Minnesota, USA, 3 centers	Recruiting
Breast cancer, head and neck cancer, rectal cancer	KD	Acute toxicity	Effect of KD on body composition in patients undergoing RT	Male and female adults	**Impact of a Ketogenic Diet Intervention During Radiotherapy on Body Composition (KETOCOMP)**: One group received a ketogenic breakfast in the form of a drink, other group followed full KD	NCT02516501	Germany, 1 center	Recruiting, some data available*
Cancer	FMD	Acute toxicity	Safety of FMD	Male and female adults	**Safety, Feasibility and Metabolic Effects of the Fasting Mimicking Diet (FMD) in Cancer Patients**	NCT03340935	Italy, 1 center	Active, not recruiting

Cancer patients receiving taxol/carboplatin treatment	FMD	Acute toxicity	Effect on nausea grade	Male and female adults	**Can Fasting Decrease the Side Effects of Chemotherapy?**	NCT04027478	California, USA, 1 center	Enrolling by invitation
Colorectal cancer	STF	Acute toxicity, Tumor growth	Effect on toxicity	Male and female adults	**Short-term Fasting as an Enhancer of Chemotherapy: Pilot Clinical Study on Colorectal Carcinoma Patients (CHEMOFAST)**: 44-48h fast, start 24h before chemotherapy	NCT04247464	Spain, 1 center	Enrolling by invitation
Glioblastoma	KD	Tumor growth	Feasibility of KD	Male and female adults	**Ketogenic Diet for Recurrent Glioblastoma (ERGO)**	NCT00575146	Germany, 2 centers	Completed*
Glioblastoma	KD + fasting	Tumor growth	Progression-free survival rates 6 months after reirradiation	Male and female adults	**Calorie-restricted, Ketogenic Diet and Transient Fasting During Reirradiation for Patients With Recurrent Glioblastoma (ERGO2)**: On day 1-3 and day 7-9, restriction of carbohydrates to < 60 g and of calories to 21-23 kcal/kg per day, on day 4-6 fasting.	NCT01754350	Germany, 2 centers	Completed, abstract oral presentation available
Glioblastoma	KD	Acute toxicity	Adherence to KD	Male and female adults (≥16 yrs)	**Ketogenic Diets as an Adjuvant Therapy in**	NCT03075514	UK, 1 center	Completed*

					**Glioblastoma (KEATING)**: Modified ketogenic diet (MKD): 80% fat and 5% carbohydrate of total kcal/day. Medium chain triglyceride (MCT) diet: 75% fat (30% of which medium chain fatty acids) and 5% carbohydrates.			
Glioblastoma	Energy-restricted KD	Tumor growth	Change in brain tumor size	Male and female adults	**Pilot Study of a Metabolic Nutritional Therapy for the Management of Primary Brain Tumors (Ketones)**	NCT01535911	Michigan, USA, 1 center	Active, not recruiting
Gynecological cancer	Fasting	Acute toxicity	QoL	Adult females	**Short-Term Fasting During Chemotherapy in Patients With Gynecological Cancer- a Randomized Controlled Cross-over Trial (FIT)**: Modified fasting (<400kcal/day) 36-48h before chemotherapy and 24h after chemotherapy	NCT01954836	Germany, 1 center	Completed*
Malignant neoplasm	Fasting	Acute toxicity	Safety and feasibility of STF	Male and female adults	**Short-Term Fasting Before Chemotherapy in**	NCT01175837	Minnesota, USA, 1 center	Completed; No results available

					**Treating Patients With Cancer**: 24 h fasting before chemotherapy, can be escalated to 36-48 h			
Non-small-cell lung cancer	KD	Tumor growth	Safety of KD during chemoradiation for lung cancer	Male and female adults	**Ketogenic Diet With Chemoradiation for Lung Cancer (KETOLUNG)**	NCT01419587	Iowa, USA, 1 center	Terminated, has results*
Non-small-cell lung cancer	FMD	Tumor growth, Acute toxicity	Effect on circulating tumor cells and its DNA damage	Male and female adults	**Fasting-mimicking Diet With Chemo-immunotherapy in Non-small Cell Lung Cancer (NSCLC)**: Plant-based FMD ~300 kcal/ fasting day	NCT03700437	Indiana, USA, 3 centers	Recruiting
Ovarian cancer, endometrial cancer	KD	NA	Changes in fasting glucose, fasting insulin and beta-hydroxybutyrate. Effect on body composition.	Adult females	**Targeted Disruption to Cancer Metabolism and Growth Through Dietary Macronutrient Modification**	NCT03171506	Alabama, USA, 1 center	Completed*
Pancreatic cancer	KD	Tumor growth	Frequency of adverse events	Male and female adults	**Ketogenic Diet With Concurrent Chemoradiation for Pancreatic Cancer (KETOPAN)**	NCT01419483	Iowa, USA, 1 center	Terminated, has results*
Prostate cancer	FMD	Acute toxicity, 3 months after FMD	Changes in body weight, blood pressure, waist circumference and cholesterol levels	Adult males	**Fasting Mimicking Diet in Prostate Cancer and Metabolic Syndrome**: 3 cycles of 5 days FMD	NCT04292041	Ireland, 1 center	Active, not recruiting

Prostate cancer	Fasting/FMD	Acute toxicity	QoL, reduction of side effects, reduction in tumor progression	Adult males	**Fasting and Nutritional Therapy in Patients With Advanced Metastatic Prostate Cancer**: 36 h before and 24 h after chemotherapy modified fasting (<400 kcal/day) or if fasting is not feasible FMD	NCT02710721	Germany, 1 center	Recruiting
Sarcoma	DR	Surgery	Effect on surgical wound complications	Male and female adults	**Effects Of Caloric Restriction On Post-Operative Complications In Sarcoma Patients Treated With Pre-Operative Radiation Therapy**: Nutritional supplement (Scandishake) providing 30% of caloric needs with 45% fat, 46% carbohydrates, 8% protein. Shake will be used 3 days before surgery.	NCT02792270	Massachusetts, USA, 1 center	Not yet recruiting
Solid tumor	Fasting	Acute toxicity	Safety/feasibility of STF, toxicity profile	Male and female adults	**Short-Term Fasting: Impact on Toxicity**: 24, 48 or 72 hours fasting before platinum-based chemotherapy	NCT00936364	California, USA, 1 center	Recruiting

Solid tumor	Fasting	Tumor growth	Response to therapy	Male and female adults	**Short-term Starvation vs. Normal Diet Before Chemotherapy of Solid Tumors**: fasting 24 h before and 6 h after chemotherapy	NCT02607826	Not provided	Unknown
Prostate cancer, endometrial cancer, breast cancer	DR	Surgery	Changes in expression of miR-21 in serum	Male and female adults	**Caloric Restriction Before Surgery in Treating Patients With Endometrial, Prostate, or Breast Cancer**: 25% DR for 3-12 weeks prior to surgery	NCT02983279	Pennsylvania, USA, 1 center	Active, not recruiting

[a] Study ID refers to clinical trials registered at clinicaltrials.gov

Supplemental table 2 – Clinical trials implementing nutritional preconditioning to study a.o. cancer prevention. DR, dietary restriction.

Subjects	Nutritional intervention	Effect on	Patient group	**Study name**, description	Study ID[a]	Location	Status
Overweight subjects	DR	Aging and incidence of age-related diseases including cancer	Males and females between 25 - 50 years	**CALERIE (PBRC, Baton Rouge) - Comprehensive Assessment of Long-Term Effects of Reducing Intake of Energy**: 6 months of DR	NCT00099151	USA, 3 centers	Completed
Healthy subjects	DR	Aging and incidence of age-related diseases including cancer	Males and females between 21 - 50 years	**CALERIE: Comprehensive Assessment of Long-Term Effects of Reducing Intake of Energy**: 25% DR for two years	NCT00427193	USA, 3 centers	Completed
Healthy, slightly overweight subjects	DR	Aging and incidence of age-related diseases including cancer	Males and females between 25 - 50 years	**CALERIE (Tufts) - Comprehensive Assessment of Long-term Effects of Reducing Intake of Energy**: 10 % or 30 % DR for one year	NCT00099099	USA, 3 centers	Completed
Overweight or obese subjects with increased risk of developing colorectal cancer	DR	Energy balance	Adult males and females >50 years	**Energy Balance Interventions for Colorectal Cancer Prevention**	NCT00653484	Tennessee, USA, 1 center	Completed
Obese subjects	DR	Inflammation levels (and potentially breast cancer risk)	Postmenopausal females	**Diet Induced Weight Loss to Reduce Inflammation in Obese Women**: diet of 800 kcal/day until 10% of initial baseline body weight was lost	NCT01699906	New York, USA, 1 center	Completed
Overweight or obese subjects	DR	Obesity-related health risks including cancer	Adult males, overweight or obese	**Nutrition and Energy Restriction for Cancer Prevention (HELENA)**: Continuous 20 % DR or 25 % DR for two days per week combined with 5 days normal intake	NCT02449148	Germany, 1 center	Completed

Subjects previously diagnosed with breast cancer	Reduced caloric intake + exercise	Breast cancer recurrence	Adult females	**Prevention of Breast Cancer Recurrence Through Weight Control, Diet, and Physical Activity Intervention (PREDICOP)**	NCT02035631	Spain, 5 centers	Not yet recruiting
Moderately overweight subjects	DR	Cancer risk	Males and females (35-65 years)	**Effect of Daily Calorie or Alternate-day Calorie Reductions on Risk for Cardiovascular Disease and Cancer (1072)**: continuous DR or alternate-day DR for 3 months	NCT00467220	California, 1 center	Completed
Subjects with metabolic syndrome	Lifestyle intervention and/or metformin	Age-related diseases including cancer	Males and females (55-74 years)	**Metformin and Dietary Restriction to Prevent Age-related Morbid Events in People With Metabolic Syndrome (MeMeMe):** lifestyle intervention consists of moderate exercise and moderate reduction of calorie and protein intake	NCT02960711	Italy, 1 center	Unknown

[a] Study ID refers to clinical trials registered at clinicaltrials.gov
